# Collaborative goal setting in palliative rehabilitation: a case report

**DOI:** 10.1186/s12904-024-01506-3

**Published:** 2024-07-19

**Authors:** Charlotte Siew Hwee Heng

**Affiliations:** HCA Hospice, Singapore, Singapore

**Keywords:** Palliative rehabilitation, Physiotherapy, Goal-setting, Patient-centred, Case report

## Abstract

**Background:**

Palliative rehabilitation amalgamates the principles of palliative care and rehabilitation to enhance patients’ quality of life by optimizing physical function and maximizing autonomy despite advancing illness. Alongside providing non-pharmacological symptom management, it emphasizes personalized goal-setting tailored to individual needs. This case illustrates the transformative impact targeted physiotherapy interventions can have on patients’ physical function, morale and motivation in spite of their expected deterioration.

**Case presentation:**

An 85-year-old male with angioimmunoblastic T cell lymphoma was admitted to home hospice care. He was referred for physiotherapy to optimize his function and mobility despite his short prognosis. A conversation guide was used throughout the duration of therapy to identify personal goals, prescribe and review the use of appropriate interventions, and discuss future therapeutic plans. Within a month he achieved his functional goals, experienced reduced physical dependency, and had increased satisfaction in his ability to participate more actively in self-care. This also resulted in significant improvements in his confidence, mood, and overall well-being. Engaging the patient actively in his care and management journey provided him with significant motivation and hope.

**Conclusion:**

The case study highlights the vital role of physiotherapists in facilitating transparent communication among patients, healthcare providers, and caregivers throughout palliative rehabilitation. Through open dialogue and utilizing conversation guides, physiotherapists help understand patient preferences, goals, and motivation. This patient-centred approach ensures that therapeutic interventions align with individual needs, enhancing overall patient care and the provision of holistic palliative care.

## Background

Patients in palliative care frequently experience marked reductions in mobility affecting not only their ability to self-care but also their mood and fulfilment of various societal roles [1]. This is often accompanied with increasing symptom burden that further limits their physical abilities, necessitating a prioritization of functional goals aligned with their energy levels and overall condition. Palliative rehabilitation is a healthcare approach that combines principles from both palliative care and rehabilitation with a primary objective of enhancing the overall quality of life of patients with life-limiting illness by optimising physical function [1] and to live as fully as possible until death [2]. This, in contrast to traditional approaches, looks at empowering individuals to maximise independence, with an emphasis on adjustments and adaptions to maximise patients’ autonomy in deciding the focus of interventions [1, 3]. It seeks to work with and empower individuals to maximise independence within the notion that any level of improvement is valuable, with emphasis on adaptations to maximise patients’ autonomy in the face of their advancing illnesses and deteriorating trajectory [3].

Implementation of palliative rehabilitation in hospice care embodies a holistic care paradigm encompassing physical, psychosocial, and spiritual components. Its practice involves tailored activities such as personalized goal-setting [4], promoting active participation in daily routines, and implementing non-pharmacological methods for managing symptoms [3] – all customised to meet the unique needs of individual patients. This process often involves detailed assessments and creating a collaborative plan that respects patient preferences while ensuring safety and feasibility [3]. The specific benefits of palliative rehabilitation are an emerging area of research in hospice and palliative care [5–7].

This case study describes the goal-setting process during physiotherapy sessions, the transformative impact of palliative rehabilitation, and the crucial role of physiotherapists in fostering shared decision-making. Collaborative goal-setting not only boosts patient morale and motivation but also enhances the effectiveness of holistic palliative care [8].

## Case presentation

Mr. H is an 85-year-old male with angioimmunoblastic T cell lymphoma with disease involving the ileum and lymph nodes. Despite multiple lines of treatment including chemotherapy, targeted therapy and immunotherapy, his disease progressed and a decision for best supportive care was made after discussion with his oncologist. He was given a prognosis of less than three months and was referred to a home hospice service for symptom management, psychosocial support and eventual terminal care. Although Mr H. maintained a regular activities routine, he experienced frequent falls prompting the hospice team to initiate a referral to physiotherapy.

On initial physiotherapy review, Mr. H was wheelchair-bound with restricted mobility from progressive lower limb weakness, compounded by a fear of further falls. He expressed dissatisfaction with his current state, indicating a -2 on the Goal Attainment Score (GAS); a measure to compare treatment outcomes while providing an overall evaluation of clinical care [9]. His Modified Barthel Index (MBI) score of 37 reflected severe dependency on caregiver assistance.

### Intervention

A discussion between his physiotherapist, Mr. H and his wife took place to explore his goals of therapy. A *conversation guide* (Table 1) was used by the physiotherapist; Step 1 allowed the identification of Mr. H’s priorities. His primary goal was to regain modified independence in mobility and was amenable to the use of a suitable walking aid. Gluteal strengthening exercises were then prescribed to initiate the reconditioning process. He was advised to continue regular home-based exercises with interval rest breaks to maximize the benefits of his exercise programme. Over subsequent reviews, Mr. H’s participation to prescribed exercises resulted in improved mobility and enhanced well-being.

**Table 1:** Conversation guide during the course of physiotherapy sessions, adapted from G-AP PC [10]

Questions to facilitate discussions during therapy	
*Step 1: Identification* • “What is most important to you right now?” • “What is something you would like to do or be better at?” *Step 2: Clarification & Negotiation* • “How does achieving … look like to you?” • “Would it be alright with you if we …” *Step 3: Reflection* • “How did you think you did?” • “What went well/not so well?” *Step 4: Planning* • “What is next?”	

Although he had resumed walking practices with the use of a walking frame to maximize independence, he had expressed a preference for the use of a walking stick instead. He valued the flexibility it offered; to be able to engage more independently in his rehabilitation, even if it meant requiring physical assistance from another person. Step 2 of the *conversation guide* was used to negotiate the appropriate choice of walking aid and establish acceptance in alignment to his wishes. This provided the physiotherapist the opportunity to offer and discuss alternative suggestions. The eventual compromise was for Mr. H to use a walking stick only with additional handhold assistance from his wife or domestic helper, to ensure a safe walking environment. This arrangement encouraged Mr. H significantly.

### Outcomes and follow-up

Within a month of enrolment in which two physiotherapy sessions were conducted, Mr. H achieved ambulation using a walking stick with minimal assistance. Significant improvement in level of dependency (MBI 37 to 53; severe to moderate) and increased satisfaction with self-performance (GAS − 2 to + 2) were observed. His first walk of more than 50 m was an emotional and remarkable moment. He shared that his achievement validated his perseverance through therapy. In response to Step 3, Mr. H reflected that this process had instilled confidence and encouraged him to continue working on his mobility and strength. He expressed gratitude to his caregivers and therapist who provided unwavering physical and emotional support.

In line with his goals of care and expectations, Mr. H progressively required less assistance with activities of daily living, actively participated in balance exercises, and continued to pace himself between exertion and rest (Step 4). His caregivers also observed an enhanced appetite and improved mood. This continued for another two months with maintenance of his gait stability and exercise tolerance. Mr. H subsequently deteriorated from disease progression, and later died from an infection.

## Discussion

Individuals with life-limiting conditions can significantly improve their physical and mental well-being through guided therapy interventions [3, 5, 11]. Traditional rehabilitative techniques such as muscle strengthening, manual therapy and physical conditioning help mitigate disease-associated musculoskeletal and cardiorespiratory complications. However, when faced with multiple concurrent concerns and limitations, prioritizing goals and planning of subsequent interventions is crucial. Palliative rehabilitation and goal-setting involve the timely identification of patient wishes and the integration of professional opinions regarding achievable outcomes. This process encompasses collaborative discussions where physiotherapists work with patients to actualize their goals while respecting individual preferences [4].

Goal-setting frameworks such as the Goal-setting and Action planning for Palliative Care (G-AP PC) model guide conversations toward outcomes that reflect meaningful change [10]. This framework leverages various theories to create effective strategies for goal-setting and achievement in palliative care. Negotiation between patients, healthcare professionals, and caregivers facilitates shared decision-making and establishes a common understanding, empowering caregivers with tools to assist patients at home. The use of a *conversation guide* allows physiotherapists to build relationships with patients and their caregivers throughout the rehabilitation process. The 4 steps of identification, negotiation, facilitating reflection and reviewing potential progression, provides structure throughout the programme, allowing a sensitive and targeted approach to effectively bring about change. In addition to improved outcomes, this strengthens the therapeutic alliance between different parties and serve as crucial emotional and psychosocial support. Goal-setting theory [12] supports how an appropriately identified and moderated goal can lead to improved persistence and effort, as well as increased goal-related performance. In addition, step 2 of the *conversation guide* incorporates the use of hope theory [13], allowing patients to develop alternative strategies in achieving their goals. By fostering confidence and allowing flexibility in decision-making, physiotherapists empower individuals to make choices that align with their values [8].

With increased accessibility to palliative care services, hospices now encounter patients with a broad spectrum of conditions at varying stages, often with the expectation that their health will deteriorate within the year. This variation presents challenges in measuring the changes or benefits resulting from rehabilitative interventions. To use existing standardized measures to monitor physical function in this group therefore poses a challenge to accurately capture and reflect the effectiveness of rehabilitation. Furthermore, the improvement of scores on these standardized measures may not result in clinically significant outcomes that the patients prioritize. Through this example, the *conversation guide* facilitated straightforward yet meaningful communication about what was important to the patient, ultimately empowering the patient to lead and direct the physiotherapy sessions.

Exercise remains valuable across the palliative care continuum, but clear and achievable goals are essential to fully utilize its benefits. Personalized goals can help patients optimize these benefits while conserving limited physiological resources. Goal-setting has the potential to empower patients to take an active role in their care, enhancing their sense of control and complementing exercise interventions to improve their quality of life [8, 10]. Although often a nuanced process that can vary greatly across populations [14], therapists need to be mindful and respectful that it may require a deeper sense of rapport and trust before patients are willing to share their goals. The use of a *conversation guide* helps clinicians understand patients’ perspectives in relation to their goals in a comprehensive but yet gentle approach. In the case of Mr. H, goal-setting kept him motivated, exercised his autonomy, and allowed his caregivers and care team to better support him throughout his remaining days.

## Conclusion

This case study underscores the importance of clear and open communication among patients, healthcare providers, and caregivers during palliative rehabilitation. In this context, the role of physiotherapists is crucial. They facilitate open dialogue to explore different rehabilitation options and guide patients and families through complexities of decision-making to optimise function alongside their expected deteriorating trajectories. Using a *conversation guide* which draws inspiration from goal-setting and hope theories, it fosters an understanding of patients’ preferences and goals, creates open channels for ongoing dialogue, and provides deeper insights into their motivations. These strategies ensure that our therapeutic interventions are both patient-centred and meaningful.

## Learning points/ take home messages

- A key component to achieving meaningful outcomes in palliative rehabilitation is patient-centred goal-setting through purposeful communication.
- Establish a platform for discussions or negotiations throughout the therapy process.
- Facilitate ongoing conversations to uncover meaning behind patients’ preferences and develop deeper understanding of their goals.

## Data Availability

The anonymized data collected are available as per documented in outcomes.
